# From Winery Waste to Biosurfactants: White Grape Pomace Fractionation, Characterization and Bioconversion Towards Sophorolipids

**DOI:** 10.3390/foods14244246

**Published:** 2025-12-10

**Authors:** Joana de Melo Martins, Stijn Bovijn, Tom Delmulle, Sofie L. De Maeseneire, Luísa S. Serafim, Sílvia Petronilho, Wim K. Soetaert

**Affiliations:** 1 Centre for Industrial Biotechnology and Biocatalysis (InBio.be), Department of Biotechnology, Faculty of Bioscience Engineering, Ghent University, Coupure Links 653, 9000 Ghent, Belgium; joana.martins@ugent.be (J.d.M.M.); stijn.bovijn@ugent.be (S.B.); tom.delmulle@ugent.be (T.D.); wim.soetaert@ugent.be (W.K.S.); 2 CICECO-Aveiro Institute of Materials, Department of Chemistry, University of Aveiro, Campus Universitário de Santiago, 3810-193 Aveiro, Portugal; luisa.serafim@ua.pt; 3 LAQV-REQUIMTE & CICECO, Department of Materials and Ceramic Engineering, University of Aveiro, Campus Universitário de Santiago, 3810-193 Aveiro, Portugal

**Keywords:** biomass, circular bioeconomy, *Starmerella bombicola*, surfactants, upcycling

## Abstract

The wine industry generates significant quantities of agro-industrial waste, with grape pomace representing its main fraction. White grape pomace (WGP), rich in fermentable sugars and lipids, can serve as a substrate for biosurfactant production. In this study, the sugar fraction of WGP was used as substrate to produce sophorolipids (SL), a class of microbial biosurfactants, by the yeast *Starmerella bombicola*. To examine efficient SL production, both a sugar source and lipid source were examined. Three lipid sources were tested: grape seed oil (GSO) extracted from WGP, spent frying oil (SFO), and rapeseed oil (RO), the later serving as a commercial reference. WGP-aqueous extraction yielded a sugar-rich fraction (WSE, 67% *w*/*w*) comprising ca. 97% carbohydrates, of which 60% were free sugars, mainly glucose and fructose. GSO accounted for 11% of the seeds’ weight and was predominantly composed of esterified linoleic (71%) and oleic (18%) acids. Bola-type SL were produced under optimized shake-flask conditions at 30 °C and 200 rpm in all WSE conditions, with SFO yielding the highest SL concentration (6.03 g/L), attributed to its oleic acid richness, and GSO yielding 3.75 g/L. This work demonstrates the potential of WGP-derived biomolecules as low-cost alternatives to first-generation substrates (commercial glucose and RO) in SL production by *S. bombicola*, contributing to the development of sustainable biosurfactants that can serve as a green alternative to petroleum-based surfactants, while reducing the environmental footprint of the wine industry.

## 1. Introduction

Grape pomace (GP) is the main winery by-product, accounting for 10–30% of the total grape mass processed during winemaking [1]. GP comprises skins (ca. 47%), stalks (ca. 28%), and seeds (ca. 25%), with its composition depending on the grape variety and processing method [2]. In fact, while red grape pomace is obtained after the fermentation of grapes, white grape pomace (WGP) is obtained after fresh grapes pressing, prior to fermentation, making WGP a more interesting source of fermentable sugars compared to red grape pomace [3,4]. Despite its abundance and its richness in carbohydrates (monosaccharides, polysaccharides, pectic polysaccharides, and lignocellulosic materials) [5,6,7], lipids [4], phenolics (predominant in red varieties), and structural proteins [8,9], GP remains underexplored. Its disposal into open fields leads to environmental pollution owing to the discharge of high organic loads, the emission of volatile organic compounds with unpleasant odors that attract pests [10], and the toxicity of phenolic compounds that decrease the soil pH, promoting antimicrobial effects [11] and increasing resistance to biological degradation [12]. Consequently, a sustainable valorization route for this by-product is highly desirable to mitigate its environmental impact and unlock its potential.

Aligning the biochemical profile of GP with the goal of reducing its disposal presents an opportunity to upcycle this winery by-product into innovative products, like biofertilizers, biofuels, biosurfactants, and cosmetics [13,14]. In the food industry, pomace components have been used to enhance food quality or enrich the nutritional content of dairy, bakery, meat, and fish products [15], or even to serve as animal feed or feed additives. Additionally, winery by-products are used as substrates for enzyme production (e.g., lipases, tannases, proteases, and xylanases) and organic acids, like citric acid [14,16]. In the energy sector, winery by-products contribute to sustainable biorefinery processes, producing low-carbon biofuels such as bioethanol, biobutanol, biogas, and bio-oil [14]. Biogas is produced through anaerobic digestion of winery by-products, offering a viable alternative to fossil-based fuels [17]. In some of these applications, GP is used as a whole, or it has been pre-treated to remove possible contaminants. However, the use of purified fractions enriched in certain compounds (like lipids or sugars) has not been described. In fact, the high content of phenolic compounds and non-fermentable sugars naturally present in GP is undesired for bioprocesses as they inhibit and limit microbial growth and production. Using fractioning, one could obtain high-purity fractions, which not only no longer contain contaminants like non-fermentable polysaccharides or phenolics, but are enriched in fermentable substrates.

In this work, a thoughtful way of fractioning WGP and driving the obtained fractions into the non-explored production of sophorolipids (SL) was presented. Biosurfactants, like SL, are biodegradable, low-toxicity molecules with anti-microbial properties, synthesized by microorganisms from renewable resources [18]. As such, they offer a green alternative to synthetic surfactants, with applications in detergents, cosmetics, oil recovery, food and beverages, and medicine, among others. SL have already been produced and commercialized as cleaning products for some years [19]. Nevertheless, their production cost remains higher than that of chemically produced surfactants, and their structural variety is comparably low, hindering their large-scale breakthrough [20]. To overcome these constraints, the usage of a low-cost substrate (to replace the commonly added glucose and rapeseed oil) and engineering the yeast strain have been suggested [21,22,23]. Utilizing side streams as substrates offers valorization, mitigating waste disposal issues while simultaneously reducing production costs, thereby enhancing both the sustainability and economic viability of a process [24,25]. Thus, in this work, a deep fractionation and characterization of WGP was performed, allowing for the extraction of a sugar–rich WGP aqueous extract (WSE) and grape seed oil (GSO), which were subsequently evaluated for bola SL production with the yeast *Starmerella bombicola*. This work contributes to the circular bioeconomy by transforming winery waste into valuable bioproducts through an integrated bioprocessing strategy, offering both environmental and economic benefits.

## 2. Materials and Methods

### 2.1. Substrates

#### 2.1.1. White Grape Pomace (WGP)

The WGP was provided by Manuel Alves Ribeiro de Almeida & Filhos company (Santa Comba Dão, Bairrada region, Portugal) and was collected after white grapes processing for sparkling wine production. Herein, the exclusive use of white grapes ensures low phenolic content that could otherwise interfere with yeast activity during biosurfactant production assays. The WGP was immediately stored at a temperature of −18 ± 1 °C, and its water content was determined, in percentage, after freeze-drying for 120 h, at 0.094 mbar and −49 °C (Labogene, Scanvac CoolSafe, Porto, Portugal), by considering the difference between the initial sample weight and the dried one (two independent replicates).

#### 2.1.2. Other Substrates

Commercial glucose (VWR, Avantor, Leuven, Belgium), oleic acid (analytical grade, 90%), and rapeseed oil (RO, commercial reference oil normally used by industry) were purchased from Sigma-Aldrich (Darmstadt, Germany) and Vandemoortele (Ghent, Belgium), respectively. Spent frying oil (SFO) was recovered from potato chip industry frying residues (“A Saloinha, Lda.”, Mafra, Portugal) by Soxhlet extraction with chloroform/methanol mixture (2:1 *V*/*V*), mainly composed of triacylglycerides (ca. 96% *w*/*w*), particularly the esterified oleic acid (81%) [26].

### 2.2. WGP Fractionation

#### 2.2.1. Sieving

After freeze-drying (48 h, 0.094 mbar, −49 °C, Labogene, Scanvac CoolSafe), the different components of WGP, particularly the stalks, seeds, skins, and dried pulp, were separated using stainless-steel sieves with different mesh sizes (4.75 mm, 2.80 mm, 2.36 mm, and 1.70 mm). At the end of the sieving process, the remaining seeds were manually separated from the skins and dried pulp fractions. The particle size distribution for the fraction containing the grape seeds (GS) and the fractions containing grape skins and dried pulp (GSP) was determined. The GS and GSP fractions were stored in a desiccator containing phosphorus pentoxide until further characterization and usage for biosurfactant production.

#### 2.2.2. Hot-Water Extraction

GSP was ground using a laboratory mill, and the obtained powder was mixed with acidified water containing acetic acid (99:1, *V*/*V*, pH 2–3). Then, a hot-water extraction was performed with a ratio of 1:6 solid/liquid for 1 h at 100 °C ± 1 °C. After cooling, the extract was filtered and centrifuged (4 °C, 15 min, 15,000 rpm) to make sure that the residue was completely separated from the water-soluble extract (WSE), before freeze-drying (48 h, 0.094 mbar, −49 °C, Labogene, Scanvac CoolSafe, Porto, Portugal). WSE had a final dry extract mass of ca. 505 g. The WSE extraction yield, expressed in percentage, was determined based on the dry extract weight in relation to the initial mass of the sample.

#### 2.2.3. Solid-Phase Extraction with C_18_ Column

The WSE was subjected to solid-phase extraction with a C_18_ cartridge (ca. 20 g, SPE-C_18_, Supelco-Discovery) to remove the phenolic compounds that can possibly inhibit the microbial growth [27]. The extraction occurred in a C_18_ column preconditioned with methanol (20 mL), followed by distilled water (20 mL) and water/acetic acid (20 mL, 99:1; *V*/*V*). Here, WSE (50 mL) was loaded into the cartridge, followed by washing with water/acetic acid (70 mL, 99/1; *V*/*V*), thus obtaining the C_18_ aqueous extract (C18A) [28]. Then, methanol/formic acid (70 mL, 99/1; *V*/*V*) was used to obtain the C_18_ methanolic extract (C18M), which corresponded to the fraction containing the WSE phenolic compounds. The column was then washed with distilled water (50 mL). C18A and C18M were concentrated in a rotavapor under reduced pressure at 40 °C ± 1 °C to remove the acidic water and methanol, respectively. C18A was then freeze-dried, and the residual methanol of C18M was evaporated using a nitrogen flux. The extraction yields of C18A and C18M, in percentage, were calculated based on the dry samples’ weight in relation to their initial mass.

#### 2.2.4. Grape Seed Oil Extraction

GS were frozen with liquid nitrogen and ground with a regular kitchen grinder. A cellulose package was filled with the GS powder (ca. 64 g) and placed inside a Soxhlet apparatus. The grape seed oil (GSO) was recovered at 70 °C, using *n*-hexane (Sigma-Aldrich), after 1.5 h (ca. 15 extraction cycles). Then, the solvent was removed from GSO with a rotary evaporator (40 °C ± 1 °C). The obtained GSO was then subjected to a gaseous nitrogen flux to evaporate the residual *n*-hexane until the sample constant weight. The recovered GSO yield of three independent replicates was determined in percentage.

### 2.3. Characterization of White Grape Pomace Fractions

The WGP, WSE C18A and C18M samples were characterized in terms of their carbohydrates, protein, and total phenolic content. Moreover, the GSO triacylglycerides content and profile were determined as fatty acid methyl esters (FAME).

#### 2.3.1. Carbohydrate Analysis

Neutral sugar content and composition were determined, in triplicate, by gas chromatography-flame ionization detection (GC-FID), after acid hydrolysis (1 M H_2_SO_4_, 2.5 h, 100 °C) and sugar residues derivatization to alditol acetates. Here, a reduction in the sugar residues with NaBH_4_ (15% *w/v* in 3 M NH_3_) occurred for 1 h at 30 °C, followed by acetylation with acetic anhydride in the presence of the catalyst 1-methymidazole, for 30 min at 30 °C. The 2-deoxyglucose (2 mg/mL) was used as the internal standard. The free sugars were determined as described for the total neutral sugars analysis, but without the hydrolysis step. For the GC-FID analysis, the samples were injected in a GC chromatograph (Clarus 500, Perkin Elmer, Waltham, MA, USA) with a capillary column DB-225 with 30 m length, 0.25 mm internal diameter, and 0.15 μm film thickness (Agilent, Santa Clara, CA, USA). The injector was kept at 220 °C and the detector at 230 °C. Hydrogen was used as carrier gas. The following temperature program was used: 1 min hold at 70 °C, increase to 170 °C at 2.0 °C/min, and then to 250 °C (5 min hold) at 16 °C/min. The identification of the sugars present in the samples was based on the retention time of the pure chemical standards. Moreover, the fructose present in each sample was quantified as the sum of mannitol and glucitol using the ratio of fructose epimerization to mannitol (43%) during the reduction step [29,30]. Uronic acids were quantified, in triplicate, by the 3-phenylphenol colorimetric method using galacturonic acid as the standard (0, 20, 40, 60, 80 μg/mL), after acid hydrolysis (1 M H_2_SO_4_, 1 h, 100 °C). The absorbance was read at 520 nm in a microplate (BioTek Instruments, Winooski, VT, USA) [30]. The carbohydrate data were expressed in terms of total polysaccharide content, estimated by the difference between the total and free sugars present in each sample under study, and in terms of the total free sugars.

#### 2.3.2. Protein Analysis

The protein content was estimated through the determination of the total nitrogen by the elemental analysis in a Truspec 630–200-200 elemental analyzer (St. Joseph, Berrien, MI, USA) with a thermal conductivity detector using two independent aliquots per sample (ca. 2 mg). The nitrogen content was converted to the protein content (% *w*/*w* dry sample) employing the 6.25 conversion factor [31].

#### 2.3.3. Total Phenolic Content

The total phenolic content was estimated by the Folin–Ciocalteu method [32]. Here, each sample (15 µL) was mixed with Folin reagent (15 µL), distilled water (60 µL), and sodium carbonate (150 µL, 7% *w*/*v*). After incubation in the dark (1 h, 60 °C), the absorbance was read in a microplate reader (BioTek Instruments, Winooski, VT, USA) at 750 nm. A calibration curve for gallic acid (0.005–0.25 mg/mL) was obtained, and the results were expressed as mg of gallic acid equivalents (GAE)/g of sample (dry weight), using three independent aliquots.

#### 2.3.4. FAME Analysis

The FAME were determined after the alkaline-catalyzed transesterification of GSO using the sample (5 g), heptadecanoate methyl ester (0.8 mL, 1.5 mg/mL in cyclohexanol), and KOH methanolic solution (0.2 mL, 2 M) as the internal standard and the catalyst, respectively. The organic phase was injected (0.5 μL) into the GC-FID (Perkin Elmer Clarus 400) equipped with a DB-FFAP column (30 m × 0.32 mm (I.D.) × 0.25 μm film thickness, J&W Scientific Inc., Folsom, CA, USA) [26,33]. The FAME compounds were identified based on the retention times of a commercial FAME mixture (C_8_–C_24_).

### 2.4. Sophorolipids Production and Characterization

#### 2.4.1. Strains

A *Starmerella bombicola* strain, previously constructed at InBio.be, with a knock-out in the acetyl transferase and lactone esterase genes, was used for the production of bola SL [34].

#### 2.4.2. Culture Media

Two media were used for the growth of *S. bombicola*: Yeast Peptone Dextrose medium (YPD; 20 g/L glucose (Cargill, Ghent, Belgium), yeast extract (10 g/L, DSM), bacto peptone (20 g/L, BD biosciences, Erembodegem, Belgium), agar (20 g/L, Biokar Diagnostics, Pantin Cedex, France)) and Lang medium (as described by Lang et al. [35]: 120 g/L glucose (Cargill), 4 g/L yeast extract (DSM), 5 g/L Na_3_C_6_H_5_O_7_·2H_2_O(Citribel, Tienen, Belgium), 1.5 g/L NH_4_Cl (VWR, Leuven, Belgium), 1 g/L KH_2_PO_4_ (Budenheim, Budenheim, Germany), 0.16 g/L K_2_HPO_4_ (Budenheim, Budeheim, Germany), 0.7 g/L MgSO_4_·7H_2_O (Sigma Aldrich), 0.5 g/L NaCl (Suprasel, Hengelo, Netherlands), 0.27 g/L CaCl_2_·2H_2_O (VWR, Leuven, Belgium). Liquid YPD (without agar) was used to grow the precultures, and liquid Lang medium was used for the *S. bombicola* production trials.

In Lang medium, different sugar sources were used to test their effect on *S. bombicola* growth and production: glucose (120 g/L), fructose (120 g/L), or WSE (120 g/L and 200 g/L). In the condition WSE_corr_, 200 g/L of WSE was used to correct the free sugar content (to the concentration of 120 g/L). Prior to autoclaving, the pH of both carbon sources and the salt solution was adjusted to 5.8. Then, sugar, salt solutions, and lipid sources were autoclaved separately for 30 min at 121 °C and 1.3 bar (Prioclave, Led Techo, Heusden-Zolder, Belgium). Sucrose solutions were sterilized by filtration with PES membranes of 0.2 μm pores (VWR).

#### 2.4.3. Production Assays

##### Growth Conditions

*S. bombicola* was inoculated from a cryovial with an inoculation needle in 5 mL YPD in a glass tube and incubated at 30 °C for 2 days on a shaker at 200 rpm. Grown cultures were then inoculated at 2% in 50 mL shake flasks with 10 mL of Lang medium with the appropriate carbon source. The flasks were incubated at 30 °C and shaken at 200 rpm for the duration of the experiment. The lipid source was added after 2 days to the shake flasks at 2% (*V*/*V*) (i.e., 17.9 g/L of oleic acid).

##### Sampling and Assay Monitoring

During the growth phase, the shake flasks were sampled to monitor biomass content, pH, sugar consumption, and SL production, in triplicate. Prior to sampling, an Eppendorf tube (1.5 mL) was weighed for every shake flask to determine the cell dry weight (CDW). These Eppendorf tubes were filled with the sample (0.5 mL), which was further processed as follows:

1. The Eppendorf tube was centrifuged for 5 min at 15,000 rpm. The residual supernatant was transferred to a new Eppendorf tube and used for pH measurement (Five easy F20, Mettler Toledo) and then divided into two Eppendorf tubes (with 0.25 mL each): one for SL analysis and one for sugar analysis.
2. The cell pellet was resuspended in NaCl solution (1 mL, 0.9%, VWR) and centrifuged again for 5 min at 15,000 rpm. The supernatant was discarded, and the Eppendorf tube was put in the oven at 70 °C to dry. After 4 days, the Eppendorf tubes with the dried cell pellet were weighed again, and the cell dry weight (CDW) was determined.

##### SL Analysis by UPLC-Evaporative Light Scattering Detector

SL were extracted by adding absolute ethanol (100% EtOH, 1 mL, Thermo Fischer Scientific, Aalst, Belgium) to the supernatant and shaking vigorously for 5 min. Next, the tubes were centrifuged for 5 min at 15,000 rpm, and the supernatant (1 mL) was transferred to a new Eppendorf tube. Samples were filtered over a 13 mm PTFE syringe filter with a pore size of 0.2 μm (Novolab, Geraardsbergen, Belgium). For the Ultra-performance liquid chromatography (UPLC) analysis of the SL, an Acquity UPLC (Waters, Antwerp, Belgium) CSH C_18_ column (130 Ä, 1.7 μm, 2.1 mm × 50 mm) was used. The sample (2 μL) was injected, the column was kept at 35 °C, and a flow rate of 0.6 mL/min was applied for 10 min/sample. A binary gradient elution system was applied, consisting of Acetic acid in ultra-pure water (0.5%, eluent A) and Acetonitrile (100%, eluent B), and performed as follows: during the first 6.8 min, the concentration of eluent B increased from 5% to 95%, after which it decreased again to 5% in 1.8 min. For the remaining 1.4 min of the sample run, the concentration of B was maintained at 5%. For the subsequent detection by the Evaporative Light Scattering Detector (Waters, Antwerp, Belgium), the nebulizer was cooled to 12 °C, and the drift tube was kept at a temperature of 50 °C. The gain was set at a value of 100.

##### Sugars Analysis by UPLC-Refractive Index Detector

For sugar analysis, each sample was acidified with H_2_SO_4_ (2M (1:1 *V*/*V*, Sigma-Aldrich)) and shaken vigorously for 5 min. Next, the tubes were centrifuged for 5 min at 15,000 rpm, and the supernatant (1 mL) was transferred to a new Eppendorf tube.

Sugars were quantified using a Waters Acquity H-class UPLC device (Waters Corporation, Milford, MA, USA) connected to an Acquity Refractive index detector (Waters Corporation, Milford, MA, USA). Metabolites were separated using a Phenomenex Rezex™ ROA-Organic Acid H+ (8%) column (Woerden, The Netherlands) operated at 60 °C. The mobile phase consisted of H_2_SO_4_ (5 mM) at a flow rate of 0.35 mL × min^− 1^. Metabolites were identified by comparing the retention times with standards. Quantification was performed by generating a calibration curve for each compound.

### 2.5. Data Analysis

The data was statistically analyzed in R (*p* < 0.05), and comparisons were performed using the *lm* and *glht* functions. Linear relationships between variables were evaluated using the lm() function in R, which fits models by ordinary least squares. Post hoc comparisons of model parameters were conducted using the glht() function from the multcomp package, which enables simultaneous testing of general linear hypotheses and controls for multiple comparisons.

## 3. Results and Discussion

### 3.1. WGP Characterization

**Figure 1:**
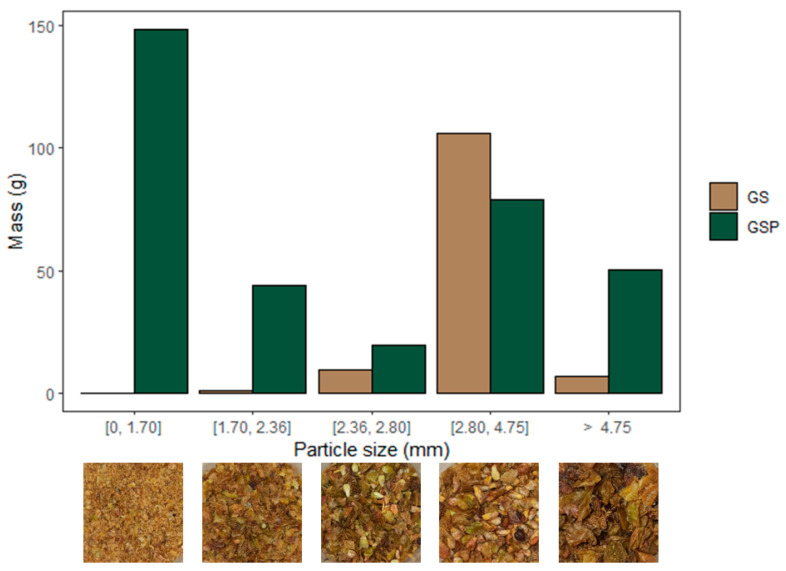
Particle size distribution in White Grape Pomace (WGP) and correspondent images of each fraction. GS: Grape seeds; GSP: Grape skins and pulp.

The WGP had a water content of ca. 71%, a value in the range reported in the literature (50–72%) [36]. When sieved, the freeze-dried WGP was composed of 74% (*w*/*w*) GSP, 23% (*w*/*w*) GS, and 3% (*w*/*w*) stalks. GSP particles exhibited heterogeneous sizes, being mainly formed by particles below 1.70 mm and bigger than 2.80 mm (Figure 1). GS size distribution was more homogeneous, being predominantly between 2.80 mm and 4.75 mm. Grape stalks were not considered further due to their low yield (3%, *w*/*w*) and potentially low free sugar content, since they are a lignocellulosic viticulture by-product known for their richness in support polysaccharides, like cellulose and hemicelluloses, which form with lignin the stalk [37].

The chemical analysis of GSP revealed that carbohydrates represent the dominant fraction of the dry sample, with a substantial proportion of free sugars and polysaccharides (60.83% and 28.36% *w*/*w*, respectively, Table 1). The sugar profile was mainly composed of glucose and fructose, while the polysaccharide fraction reflected the presence of glucose-rich structures together with uronic acids and minor amounts of arabinose. This polysaccharide composition is consistent with the GP carbohydrate profile, particularly with pectic polysaccharides and cellulose/hemicelluloses from the pulp and cell wall material [38].

**Figure 2:**
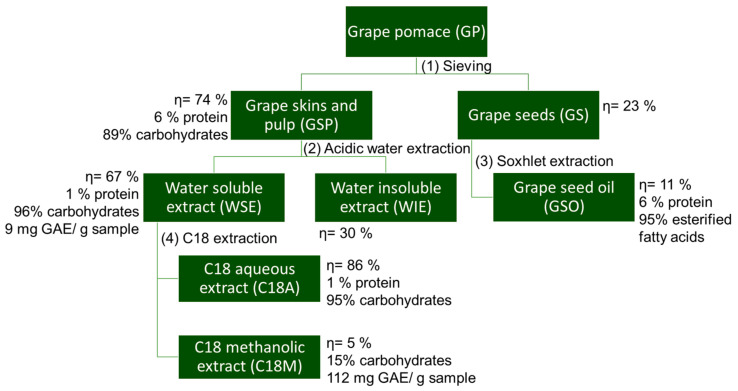
Fractionation of White Grape Pomace (WGP). Summary of the yields (η), chemical composition of WGP extracts, including total carbohydrate (%), protein (%), and phenolic content (mg gallic acid equivalents (mg GAE)/g of dry weight sample).

After a GSP hot-water extraction, two fractions were obtained (Figure 2): a water-soluble extract (WSE) and a water-insoluble extract (WIE, the precipitate from the centrifugation step). WSE and WIE represent 66.51% and 29.78% of the dry GSP weight, respectively. WSE consists of carbohydrates (96.50%, *w*/*w*), comprising 59.64% free sugars and 36.96% polysaccharides, with a minor protein content (1.67%, *w*/*w*). Moreover, the WSE had a total phenolic content of 9.2 mg GAE/g sample, which is comparable to values reported for other freeze-dried WGP extracts (4.55–31.13 mg GAE/g) [39].

When the WSE was subjected to a solid-phase extraction using a C_18_ cartridge to remove the phenolic compounds present (Figure 2), two extracts were obtained, one corresponding to the non-retained aqueous fraction (C18A) and the other related to the retained fraction (C18M) recovered with methanol and rich in phenolics (Table 1). C18A corresponded to 86.15% of the dry WSE weight, being mainly constituted by carbohydrates (94.77% *w*/*w*) and protein (1.28% *w*/*w*). On the other hand, C18M represented 5.47% of the dry WSE weight and was constituted of only 15.10% of polysaccharides and 111.99 mg GAE/g sample of phenolic compounds. The polysaccharides identified in the C18M fraction suggest the presence of sugar-polyphenol complexes through covalent bonding, similarly to what was observed with apple pomace [28,40]. Due to high mol% of glucose (Table 1), it can be suggested that the phenolic compounds of C18M were retained due to their covalent interaction with glucans, as reported in wine polyphenols–polysaccharides [41].

Regarding GSO, which was recovered from GS after Soxhlet extraction with *n*-hexane, it corresponds to ca. 11% of the dry mass of GS, in line with the 6% to 22% range reported for oil recovered from other GS [42]. GSO was constituted by 952.5 mg of FAME/g sample, mainly long-chain unsaturated fatty acids (89% of the total GSO mass), such as linoleic (71%) and oleic (18%) acids (Figure S1). These values were in line with the literature for linoleic and oleic acids of GSO (58–78% and 12–28%, respectively). In addition, the saturated fatty acids corresponded to 11% of the total GSO mass, corresponding to 6% and 5% of palmitic and stearic acids, respectively, in accordance with the literature [43].

### 3.2. Sophorolipids Production

To identify an effective valorization route for the WGP, both WSE and GSO were tested as substrates to produce SL using the yeast *S. bombicola*. These fractions were selected due to their richness in sugars and lipids, respectively, as well as their potential to deliver higher yields with fewer fractionation steps. A genetically engineered strain was used with knock-outs in both the acetyl transferase and the lactone esterase genes, which results in a strain with a more uniform production profile, yielding predominantly non-acetylated bola SL [34]. Such bola SL are more water soluble, facilitating quantification to wild-type SL, and can lower surface tension to 36.4 mN/m [44]. In brief, SL biosynthesis began with the uptake or synthesis of a fatty acid, followed by hydroxylation via cytochrome P450 monooxygenase. Two glycosyltransferases then add glucose moieties to both sides of the hydroxy fatty acid, after which acetylation may occur through an acetyl transferase. The resulting bola SL were secreted by a transporter. In strains where lactone esterase is active, one sophorose unit is cleaved, and intramolecular transesterification produces lactonic SL [34].

#### 3.2.1. Influence of the Lipid Source

In addition to GSO, three more lipid sources, including oleic acid (OA), rapeseed oil (RO), and spent frying oil (SFO), were used to evaluate and compare their efficiency for bola SL production. These lipid-based substrates were selected since GSO was observed to be a source of esterified linoleic and oleic acids. OA is the preferred substrate for *S. bombicola* [45], RO is normally used as the industrial standard [46], and SFO has been gaining popularity as a second-generation feedstock, for amongst others, SL production [47].

**Figure 3:**
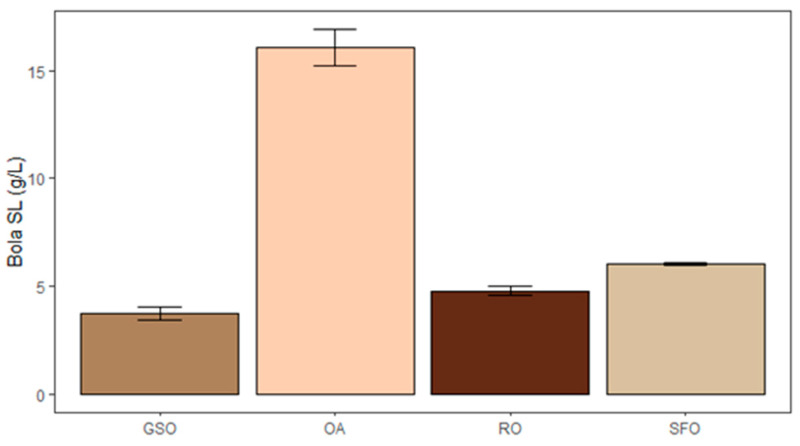
Production of bola sophorolipids by *S. bombicola* when using grape seed oil (GSO), oleic acid (OA), rapeseed oil (RO), and spent frying oil (SFO) as lipid-based substrates.

The higher concentration of SL (Figure 3) was obtained with OA (16.09 g/L bola SL), followed by SFO (6.03 g/L bola SL), RO (4.78 g/L bola SL), and GSO (3.75 g/L bola SL). This can be explained by the preference of *S. bombicola* for oleic acid [45] and the relative content thereof in the substrates. Moreover, in SFO, RO, and GSO, fatty acids, like oleic acid, are present in the form of triacylglycerides, which might slow down the uptake of substrate a bit. Still, at an industrial scale, lipid-rich raw materials like RO are preferably used rather than pure oleic acid, due to their lower cost. Depending on the source, the oleic acid content in RO can vary, yet it seldom exceeds 60% [48]. If taking this into account, both SFO and GSO can be considered promising lipid sources to produce SL, since their production did not differ significantly from the RO condition (*p* > 0.05). This means that SFO and GSO are viable alternatives as substrates for SL production, in comparison with the industrial standard. Depending on cost and local availability, these substrates will likely be cheaper and provide a more sustainable alternative to the commonly used rapeseed oil, as they are waste-derived. To increase production to levels comparable to the ones obtained with pure oleic acid, it might be interesting to see if other SL-producing *Starmerella* species have a higher acceptance for the fatty acid mixtures present in these oils. A chemical saturation could also be equated to convert linoleic acid to oleic acid, but this will further increase the operation costs.

#### 3.2.2. Influence of Sugar Source

**Figure 4:**
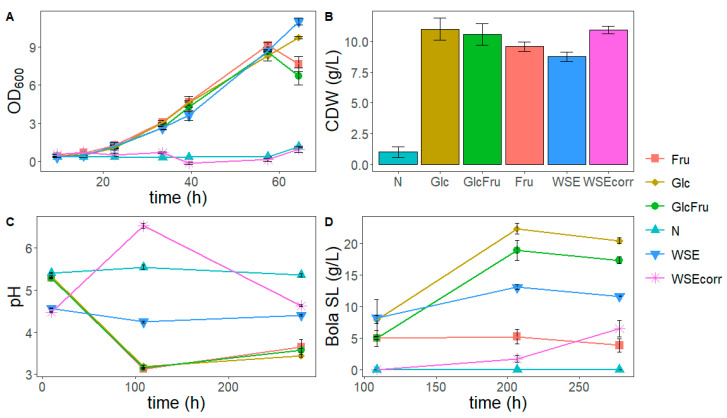
Summary of parameters analyzed for the study of the influence of sugar source. (**A**) Optical density at 600 nm (OD_600_) profile of the exponential growth phase; (**B**) final cell dry weight (CDW) concentrations (g/L), obtained at 10 days of the growth trial with the different conditions tested; (**C**) pH profile of the growth trial; (**D**) production profile of sophorolipids during the trial. N: condition with no sugar source (negative control), Fru: condition with 120 g/L fructose, Glc: condition with 120 g/L glucose, GlcFru: 60g/L of both glucose and fructose, WSE: 120 g/L of WGP aqueous extract (WSE), corresponding to 72 g/L of glucose and fructose, WSEcorr: 200 g/L of WSE, corresponding to 120 g/L of glucose and fructose.

To compare the consumption of different sugar sources and to determine the viability of using the WSE to replace first-generation sugars, a production trial on shake flasks was set up. As a lipid source, the OA standard (17.9 g/L) was fixed for all conditions. Regarding the sugar source, several conditions were tested: glucose (Glc, 120 g/L), fructose (Fru, 120 g/L), glucose and fructose mix (GlcFru, 60 g/L of each sugar), WSE (120 g/L, which corresponds to 72 g/L of glucose and fructose), and WSE_corr_. The latter condition was added to ensure the final glucose and fructose content of around 120 g/L, considering that 59.64% of the total mass are free sugars (Table 1). A negative control (N) without a sugar source was also considered. Growth (cell dry weight, CDW, and optical density, OD_600_), pH profile, sugar consumption, and SL production were evaluated (Figure 4).

The CDW concentrations after 10 days of growth trials are shown in Figure 4B. The negative control N revealed some residual growth (*p* < 0.05), which can be attributed to, for example, the consumption of carbon present in the yeast extract or the citric acid in the Lang medium. The conditions Fru, GlcFru, and the ones with WSE did not significantly differ from the positive control (Glc) (*p* > 0.05). Although the final CDW in the WSE_corr_ was similar to the positive control, the growth in this sugar source started much later, as can be seen in the OD_600_ profile of the first 65h of the growth trial (Figure 4A). This can possibly be attributed to a lower oxygen transfer, as it was observed that the medium becomes quite viscous when 200 g/L of WSE was added. The culture might have gone (partially) anaerobic as traces of ethanol and mannitol were produced (below the limit of quantification). In addition to this, a longer lag phase can also be attributed to a higher concentration of one or more inhibiting compounds, like phenolic compounds (Table 1), in the WSE_corr_, which required some adaptation time in these higher concentrations.

Regarding the pH (Figure 4C), it was set at 5.8 for all media before autoclaving. In the N condition, the pH remains stable during the whole experiment, even with the addition of OA before the production phase, which is consistent with the very limited growth observed in N and the buffering capacity of the medium. For the Glc, Fru, and GlcFru trials, there is a drop in the pH values to below 3.5. This is commonly observed during the production of SL, where the medium is controlled at pH 4 in bioreactors [46]. For WSE, at the first measuring point, after 9.5 h, the shake flasks already have a lower pH compared to the other conditions, remaining almost stable throughout the experiment. This behavior may be related to the WSE viscosity, possibly due to its high sugar content and the presence of soluble oligo- and polysaccharides derived from as pectic polysaccharides and hemicelluloses (Table 1), together with its buffering capacity around the pKa of acetic acid (added during WSE extraction, pKa of 4.76). Possible reactions during autoclaving cannot be excluded, as the pH was initially adjusted to 5.8. Regarding the WSE_corr_ condition, the pH value increased in the first 100 h of the trial, before going down again at the end of the experiment. The reason behind this increase can be related to the long lag phase observed in this condition (Figure 4A), where there might have been limited oxygen, which could have caused the yeast to metabolize the available compounds in a distinct way, or some of the leftover acetic acid evaporate from the extraction.

The SL concentrations obtained during the trials with the different media are shown in Figure 4D. The negative control did not produce detectable amounts of SL since the Lang medium did not provide enough carbon and, therefore, only allowed very limited growth (Figure 4D). When glucose was the only carbon source, the highest bola SL production was obtained (20.45 g/L). This value is slightly higher than in the lipid source experiment, as here 100 mL shake flasks instead of 50 mL shake flasks were used.

**Figure 5:**
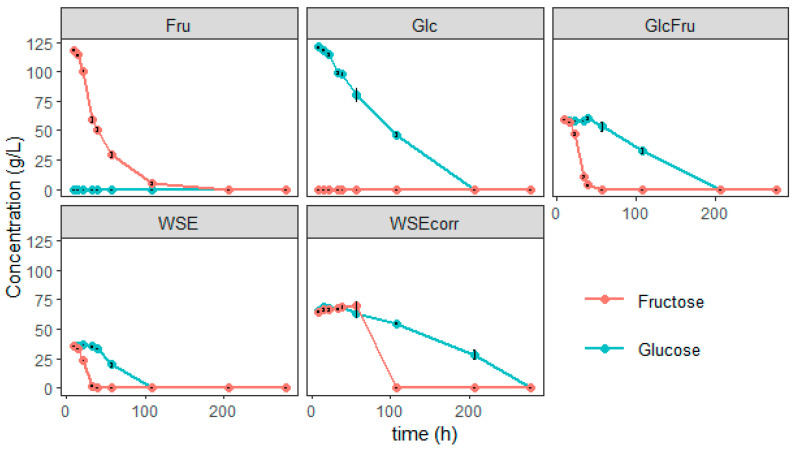
Glucose and fructose concentrations during the growth trial with *S. bombicola* on different media; Fru: condition with 120 g/L fructose, Glc: condition with 120 g/L glucose, GlcFru: 60 g/L of both glucose and fructose, WSE: 120 g/L of WGP aqueous extract (WSE), corresponding to 72 g/L of glucose and fructose, WSEcorr: 200 g/L of WSE, corresponding to 120 g/L of glucose and fructose.

The GlcFru condition exhibited the second-highest concentration (17.31 g/L) (*p* > 0.05), as enough glucose was still present for the production phase for incorporation into the bola SL [34]. This is due to the fructophilic growth behavior of *S. bombicola*, where fructose is first consumed (Figure 5) [49].

When only fructose is present, the production of bola SL is low (3.84 g/L). For growth, *Starmerella* species are fructophilic, meaning they have a preference for fructose over glucose [49]. This is also clear from these experiments in which the fructose is clearly consumed before glucose (Figure 5). However, for SL production, the opposite was observed since the glucose is preferred (which is coherent since SL need glucose units and not fructose units). In the GlcFru condition, the fructose is first consumed for growth, so at the beginning of the production phase, there is still enough glucose present for efficient production.

In all conditions, it is clear that, when present, the fructose is consumed first (Figure 5) and both glucose and fructose are completely gone at the end of the experiments. In the conditions where fructose is present, about 60 g/L is consumed before the addition of oleic acid. However, the titer of the bola is quite different and remarkably lower, prompting the question as to where all the other carbon went. To tackle this, more research needs to be conducted.

In the WSE condition, the soluble sugars consist predominantly of glucose and fructose in almost an even ratio. This condition is thus like the GlcFru condition, but with a lower absolute amount of free sugars present and some other grape components as well. Since WSE is a replacement for the sugar source, initially, the WSE was added with the same mass as the other sugars (120 g/L), yet the WSE condition has a lower total amount of free sugars (glucose and fructose, which can be consumed by the *S. bombicola*) because only 59.64% of its mass consists of free sugars. Therefore, the free sugar content added was 71.6 g/L. The end titer was 11.63 g/L of bola sophorolipids, which is a little more than 59.64% of the glucose + fructose condition (part of the bola SL mass also comes from the oleic acid). If taking this into account, the WSE produces a predicted content of SL in comparison with the GlcFru condition, and it seems as a viable alternative feedstock to make SL production more sustainable.

In the last condition, WSE_corr_, an amount of WSE was added so the total amount of free sugars was the same as in the other conditions. Surprisingly, the titer of bola SL was even lower (6.51 g/L) than in the WSE condition. However, it is important to note that the growth in this condition was remarkably slower. This can possibly be attributed to the lower oxygen transfer as the medium became quite viscous when this amount of WSE is added. Other factors, such as higher osmotic pressure and the presence of inhibitory compounds (e.g., acetic acid), may also contribute to the slower growth and reduced yields. These mechanisms could act synergistically, although further experiments would be required to confirm their relative impact. The production started later, possibly due to the oxygen limitation, which might also explain the lower titers. The production could therefore still have been ongoing, although there was no more glucose in the last point. Scaling the process up to a bioreactor with stirring, aeration, and pH control could solve these problems and bring the SL titer to the expected values. If not, the WSE at lower concentrations can be used for the growth phase in a fed-batch reactor and then glucose (either first generation or derived from another side stream) can be fed to counter the problem. By starting in a fed-batch reactor with a lower amount of WSE, the viscosity problems might also be solved. The WSE could be used as feed as well and thus has many possibilities to be used for sustainable SL production. *S. bombicola* fermentations have been performed at a larger scale for both the wild-type and novel glycolipids with engineered strains. A continuous process for the production of bola SL with a mean productivity of 0.56 g L^−1^ h^−1^ has been described [50] and would form a solid base for the scaling-up of WSE-based SL.

## 4. Conclusions

The holistic fractionation of WGP successfully yielded carbohydrate and lipid-enriched extracts (WSE and GSO, respectively) suitable for microbial bioconversion. WSE and GSO exhibited high purity (ca. 97% and ca. 95% (*w*/*w*) of carbohydrates and triacylglycerides, respectively), supporting their effective use as substrates for SL production by *S. bombicola*. Bola SL production was established on WSE, while GSO demonstrated potential as a sustainable alternative to replace the industrial reference (RO), along with SFO, thus providing a green alternative to petroleum-based surfactants widely used in detergents and cosmetics. These findings highlight the potential of WGP-derived waste streams as low-cost renewable feedstocks for sustainable biosurfactants production. While small-scale Erlenmeyer experiments cannot be directly compared to optimized bioreactor processes, the results provide a proof-of-concept that supports further development. Future work should address process limitations, scale-up strategies, and economic viability. Moreover, the insoluble pomace residue could also be valorized through its insoluble fiber and protein fractions, as food additives or bioenergy production to support an integrated valorization strategy. Overall, this study advances circular bioeconomy approaches and contributes to reducing the environmental footprint of the wine industry.

## Figures and Tables

**Table 1:** Yield, monosaccharide, carbohydrate, protein, and phenolic composition (mg gallic acid equivalents (GAE)/g of dry weight sample) of White Grape Pomace (WGP) extracts (Grape skins and pulp (GSP), WGP aqueous extract (WSE), C18 aqueous extraction (C18A), and C18 methanolic extraction (C18M)).

	Yield (%, *w*/*w*) *		Monosaccharide Composition (molar %)					Total Carbohydrates (%, *w*/*w*)		Total Protein (%, *w*/*w*)	Total Phenolic Content (mg GAE/g)
			Ara	Fru ^1^	Glc	Gal	UA				
GSP	29.35	Poly	1	0	57	tr	41	28.36	89.19	5.89	n.d.
		Free		48	52			60.83			
WSE	66.51	Poly	1	0	82	0	17	36.86	96.50	1.67	9.17
		Free		51	49			59.64			
C18A	86.15	Poly	0	0	85	0	15	32.43	94.76	1.28	tr
		Free		47	53			62.33			
C18M	5.47	Poly	7	0	85	tr	8	15.10	15.10	n.d.	111.99
		Free		48	52			tr			

* The GSP yield was expressed as the % of its dried weight after the separation of seeds and stalks, WSE yield was expressed as the % of GSP, and the yield of C18A and C18M was expressed as the % of WSE. The protein from WIE was analyzed to estimate the protein of GSP (sum of WIE and WSE protein content). WIE was the precipitate from the centrifugation step. Poly—polysaccharides, Ara—arabinose, Fru—fructose, Glc—glucose, Gal—galactose, and UA—uronic acids; n.d.—not determined, tr—traces, GAE—gallic acid equivalents. ^1^ Fru was estimated as the sum of mannitol and glucitol using its epimerization ratio during the reduction step [29,30].

## Data Availability

The original contributions presented in this study are included in the article/Supplementary Material. Further inquiries can be directed to the corresponding authors.

## Supplementary Materials

The following supporting information can be downloaded at: https://www.mdpi.com/article/10.3390/foods14244246/s1, Figure S1: Esterified fatty acids composition (determined as FAME) of GSO.

## Abbreviations

The following abbreviations are used in this manuscript:

C18A	C18 aqueous extraction
C18M	C18 methanolic extraction
CDW	Cell dry weight
FAME	Fatty acid methyl esters
Fru	Fructose
GAE	Gallic acid equivalents
Glc	Glucose
GlcFru	Glucose-fructose mix condition
GP	Grape pomace
GS	Grape seeds
GSO	Grape seed oil
GSP	Grape skins and pulp
OA	Oleic acid
OD_600_	Optical Density at 600 nm
RO	Rapeseed oil
SFO	Spent frying oil
SL	Sophorolipids
WGP	White grape pomace
WIE	Water-insoluble extract
WSE	WGP aqueous extract
WSE_corr_	WGP aqueous extract corrected to 120 g/L free sugars
YPD	Yeast peptone dextrose medium

## References

[1] Rani J., Indrajeet, Rautela A., Kumar S., Krishnaraj Rathinam N., Sani R.K. (2020). Chapter 4—Biovalorization of Winery Industry Waste to Produce Value-Added Products. Biovalorisation of Wastes to Renewable Chemicals and Biofuels.

[2] Kokkinomagoulos E., Kandylis P. (2023). Grape pomace, an undervalued by-product: Industrial reutilization within a circular economy vision. Rev. Environ. Sci. Biotechnol..

[3] Dwyer K., Hosseinian F., Rod M. (2014). The Market Potential of Grape Waste Alternatives. J. Food Res..

[4] Rodrigues R.P., Gando-Ferreira L.M., Quina M.J. (2022). Increasing Value of Winery Residues through Integrated Biorefinery Processes: A Review. Molecules.

[5] Sirohi R., Tarafdar A., Singh S., Negi T., Gaur V.K., Gnansounou E., Bharathiraja B. (2020). Green Processing and Biotechnological Potential of Grape Pomace: Current Trends and Opportunities for Sustainable Biorefinery. Bioresour. Technol..

[6] Corbin K.R., Hsieh Y.S.Y., Betts N.S., Byrt C.S., Henderson M., Stork J., DeBolt S., Fincher G.B., Burton R.A. (2015). Grape Marc as a Source of Carbohydrates for Bioethanol: Chemical Composition, Pre-Treatment and Saccharification. Bioresour. Technol..

[7] Chamorro S., Viveros A., Alvarez I., Vega E., Brenes A. (2012). Changes in Polyphenol and Polysaccharide Content of Grape Seed Extract and Grape Pomace after Enzymatic Treatment. Food Chem..

[8] González-Centeno M.R., Rosselló C., Simal S., Garau M.C., López F., Femenia A. (2010). Physico-Chemical Properties of Cell Wall Materials Obtained from Ten Grape Varieties and Their Byproducts: Grape Pomaces and Stems. LWT—Food Sci. Technol..

[9] Baruwati B., Varma R.S. (2009). High Value Products from Waste: Grape Pomace Extract—A Three-in-One Package for the Synthesis of Metal Nanoparticles. ChemSusChem.

[10] Christ K.L., Burritt R.L. (2013). Critical Environmental Concerns in Wine Production: An Integrative Review. J. Clean. Prod..

[11] Takó M., Kerekes E.B., Zambrano C., Kotogán A., Papp T., Krisch J., Vágvölgyi C. (2020). Plant Phenolics and Phenolic-Enriched Extracts as Antimicrobial Agents against Food-Contaminating Microorganisms. Antioxidants.

[12] Bustamante M.A., Moral R., Paredes C., Pérez-Espinosa A., Moreno-Caselles J., Pérez-Murcia M.D. (2008). Agrochemical Characterisation of the Solid By-Products and Residues from the Winery and Distillery Industry. Waste Manag..

[13] Rivera O.M.P., Moldes A.B., Torrado A.M., Domínguez J.M. (2007). Lactic Acid and Biosurfactants Production from Hydrolyzed Distilled Grape Marc. Process Biochem..

[14] Bharathiraja B., Iyyappan J., Jayamuthunagai J., Kumar R.P., Sirohi R., Gnansounou E., Pandey A. (2020). Critical Review on Bioconversion of Winery Wastes into Value-Added Products. Ind. Crops Prod..

[15] Zhu F., Du B., Zheng L., Li J. (2015). Advance on the Bioactivity and Potential Applications of Dietary Fibre from Grape Pomace. Food Chem..

[16] Papadaki E., Mantzouridou F.T. (2019). Citric Acid Production from the Integration of Spanish-Style Green Olive Processing Wastewaters with White Grape Pomace by *Aspergillus niger*. Bioresour. Technol..

[17] Mak T.M.W., Xiong X., Tsang D.C.W., Yu I.K.M., Poon C.S. (2020). Sustainable Food Waste Management towards Circular Bioeconomy: Policy Review, Limitations and Opportunities. Bioresour. Technol..

[18] Delbeke E.I.P., Everaert J., Lozach O., Le Gall T., Berchel M., Montier T., Jaffrès P.-A., Rigole P., Coenye T., Brennich M. (2019). Lipid-Based Quaternary Ammonium Sophorolipid Amphiphiles with Antimicrobial and Transfection Activities. ChemSusChem.

[19] Dierickx S., Castelein M., Remmery J., De Clercq V., Lodens S., Baccile N., De Maeseneire S.L., Roelants S.L.K.W., Soetaert W.K. (2022). From Bumblebee to Bioeconomy: Recent Developments and Perspectives for Sophorolipid Biosynthesis. Biotechnol. Adv..

[20] Wang H., Kaur G., To M.H., Roelants S.L.K.W., Patria R.D., Soetaert W., Lin C.S.K. (2020). Efficient in-Situ Separation Design for Long-Term Sophorolipids Fermentation with High Productivity. J. Clean. Prod..

[21] Wang H., Roelants S.L., To M.H., Patria R.D., Kaur G., Lau N.S., Lau C.Y., Van Bogaert I.N., Soetaert W., Lin C.S. (2019). *Starmerella bombicola*: Recent Advances on Sophorolipid Production and Prospects of Waste Stream Utilization. J. Chem. Technol. Biotechnol..

[22] Nitschke M., Ferraz C., Pastore G.M. (2004). Selection of Microorganisms for Biosurfactant Production Using Agroindustrial Wastes. Braz. J. Microbiol..

[23] Makkar R.S., Cameotra S.S., Banat I.M. (2011). Advances in Utilization of Renewable Substrates for Biosurfactant Production. AMB Express.

[24] Baccile N., Babonneau F., Banat I.M., Ciesielska K., Cuvier A.-S., Devreese B., Everaert B., Lydon H., Marchant R., Mitchell C.A. (2017). Development of a Cradle-to-Grave Approach for Acetylated Acidic Sophorolipid Biosurfactants. ACS Sustain. Chem. Eng..

[25] Kaur G., Wang H., To M.H., Roelants S.L.K.W., Soetaert W., Lin C.S.K. (2019). Efficient Sophorolipids Production Using Food Waste. J. Clean. Prod..

[26] Petronilho S., Oliveira A., Domingues M.R., Nunes F.M., Coimbra M.A., Gonçalves I. (2021). Hydrophobic Starch-Based Films Using Potato Washing Slurries and Spent Frying Oil. Foods.

[27] Adeboye P.T., Bettiga M., Olsson L. (2014). The Chemical Nature of Phenolic Compounds Determines Their Toxicity and Induces Distinct Physiological Responses in Saccharomyces Cerevisiae in Lignocellulose Hydrolysates. AMB Express.

[28] Fernandes P.A.R., Le Bourvellec C., Renard C.M.G.C., Nunes F.M., Bastos R., Coelho E., Wessel D.F., Coimbra M.A., Cardoso S.M. (2019). Revisiting the Chemistry of Apple Pomace Polyphenols. Food Chem..

[29] Brunton N.P., Gormley T.R., Murray B. (2007). Use of the Alditol Acetate Derivatisation for the Analysis of Reducing Sugars in Potato Tubers. Food Chem..

[30] Petronilho S., Navega J., Pereira C., Almeida A., Siopa J., Nunes F.M., Coimbra M.A., Passos C.P. (2023). Bioactive Properties of Instant Chicory Melanoidins and Their Relevance as Health Promoting Food Ingredients. Foods.

[31] Sosulski F.W., Imafidon G.I. (1990). Amino Acid Composition and Nitrogen-to-Protein Conversion Factors for Animal and Plant Foods. J. Agric. Food Chem..

[32] Singleton V.L., Rossi J.A. (1965). Colorimetry of Total Phenolics with Phosphomolybdic-Phosphotungstic Acid Reagents. Am. J. Enol. Vitic..

[33] Gonçalves I., Lopes J., Barra A., Hernández D., Nunes C., Kapusniak K., Kapusniak J., Evtyugin D.V., Lopes da Silva J.A., Ferreira P. (2020). Tailoring the Surface Properties and Flexibility of Starch-Based Films Using Oil and Waxes Recovered from Potato Chips Byproducts. Int. J. Biol. Macromol..

[34] Roelants S.L.K.W., Bovijn S., Bytyqi E., de Fooz N., Luyten G., Castelein M., Van de Craen T., Diao Z., Maes K., Delmulle T. (2024). Bubbling Insights: Unveiling the True Sophorolipid Biosynthetic Pathway by *Starmerella bombicola*. Biotechnol. Biofuels.

[35] Lang S., Brakemeier A., Heckmann R., Spöckner S., Rau U. (2000). Production of Native and Modified Sophorose Lipids. Chim. Oggi.

[36] Spinei M., Oroian M. (2021). The Potential of Grape Pomace Varieties as a Dietary Source of Pectic Substances. Foods.

[37] Chen H., Chen H. (2014). Chemical Composition and Structure of Natural Lignocellulose. Biotechnology of Lignocellulose: Theory and Practice.

[38] Rivas M.Á., Casquete R., Córdoba M.d.G., Ruíz-Moyano S., Benito M.J., Pérez-Nevado F., Martín A. (2021). Chemical Composition and Functional Properties of Dietary Fibre Concentrates from Winemaking By-Products: Skins, Stems and Lees. Foods.

[39] José Jara-Palacios M., Hernanz D., Luisa Escudero-Gilete M., Heredia F.J. (2014). Antioxidant Potential of White Grape Pomaces: Phenolic Composition and Antioxidant Capacity Measured by Spectrophotometric and Cyclic Voltammetry Methods. Food Res. Int..

[40] Fernandes P.A.R., Ferreira S.S., Bastos R., Ferreira I., Cruz M.T., Pinto A., Coelho E., Passos C.P., Coimbra M.A., Cardoso S.M. (2019). Apple Pomace Extract as a Sustainable Food Ingredient. Antioxidants.

[41] Gonçalves F.J., Fernandes P.A.R., Wessel D.F., Cardoso S.M., Rocha S.M., Coimbra M.A. (2018). Interaction of Wine Mannoproteins and Arabinogalactans with Anthocyanins. Food Chem..

[42] Shinagawa F.B., de Santana F.C., Torres L.R.O., Mancini-Filho J. (2015). Grape Seed Oil: A Potential Functional Food?. Food Sci. Technol..

[43] CODEX STAN 210-1999 Standard for Named Vegetable Oils-CAC Standards-International-Food Laws & Regulations-Documents-Global FoodMate. http://files.foodmate.com/2013/files_1054.html.

[44] Van Bogaert I.N.A., Buyst D., Martins J.C., Roelants S.L.K.W., Soetaert W.K. (2016). Synthesis of Bolaform Biosurfactants by an Engineered *Starmerella bombicola* Yeast. Biotechnol. Bioeng..

[45] Huang F.-C., Peter A., Schwab W. (2014). Expression and Characterization of CYP52 Genes Involved in the Biosynthesis of Sophorolipid and Alkane Metabolism from *Starmerella bombicola*. Appl. Environ. Microbiol..

[46] Van Bogaert I.N.A., Saerens K., De Muynck C., Develter D., Soetaert W., Vandamme E.J. (2007). Microbial Production and Application of Sophorolipids. Appl. Microbiol. Biotechnol..

[47] Hirata Y., Igarashi K., Ueda A., Quan G.L. (2021). Enhanced Sophorolipid Production and Effective Conversion of Waste Frying Oil Using Dual Lipophilic Substrates. Biosci. Biotechnol. Biochem..

[48] Gunstone F. (2009). Rapeseed and Canola Oil: Production, Processing, Properties and Uses.

[49] Gonçalves C., Wisecaver J.H., Kominek J., Oom M.S., Leandro M.J., Shen X.-X., Opulente D.A., Zhou X., Peris D., Kurtzman C.P. (2018). Evidence for Loss and Reacquisition of Alcoholic Fermentation in a Fructophilic Yeast Lineage. eLife.

[50] Dierickx S., Maes K., Roelants S.L.K.W., Pomian B., Van Meulebroek L., De Maeseneire S.L., Vanhaecke L., Soetaert W.K. (2022). A Multi-Omics Study to Boost Continuous Bolaform Sophorolipid Production. New Biotechnol..

