# Supplementary material for: From Winery Waste to Biosurfactants: White Grape Pomace Fractionation, Characterization and Bioconversion Towards Sophorolipids
Source: Foods. 2025 Dec 10;14(24):4246. doi: 10.3390/foods14244246 (PMC12731870; doi:10.3390/foods14244246)
Supplement: Supplementary file 1 [file foods-14-04246-s001.zip › foods-3997704-supplementary.pdf]

From Winery Waste to Biosurfactants: White Grape Pomace Fractionation, Characterization and Bioconversion Towards Sophorolipids

Joana de Melo Martins [1,†], Stijn Bovijn [1,†], Tom Delmulle [1], Sofie L. De Maeseneire [1,*], Luísa S. Serafim [2], Sílvia Petronilho [3,*] and Wim K. Soetaert [1]

[1] Centre for Industrial Biotechnology and Biocatalysis (InBio.be), Department of Biotechnology, Faculty of Bioscience Engineering, Ghent University, Coupure Links 653, 9000 Ghent, Belgium

[2] CICECO-Aveiro Institute of Materials, Department of Chemistry, University of Aveiro, Campus Universitário de Santiago, 3810-193 Aveiro, Portugal

[3] LAQV-REQUIMTE & CICECO, Department of Materials and Ceramic Engineering, University of Aveiro, Campus Universitário de Santiago, 3810-193 Aveiro, Portugal

* Authors to whom correspondence should be addressed.

† Both authors contributed equally to this work.

Correspondence: Sofie.DeMaeseneire@ugent.be and silviapetronilho@ua.pt

Supplementary Figure

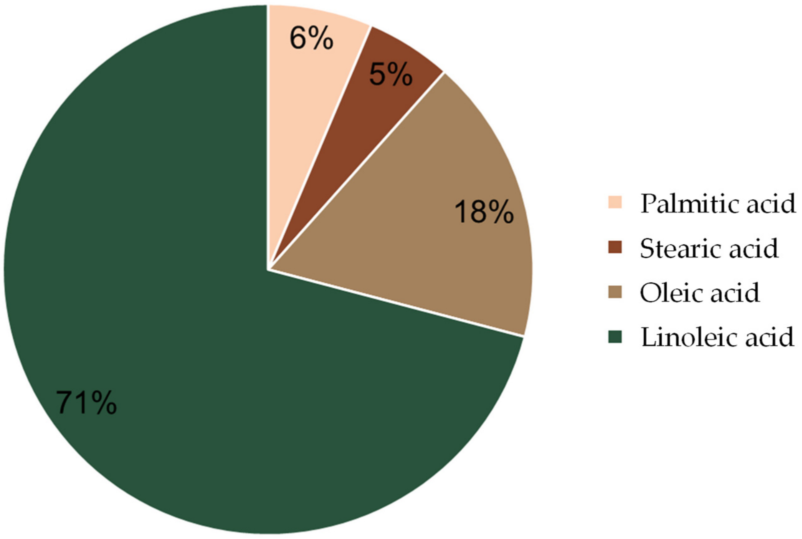

Figure S1: Esterified fatty acids composition (determined as Fatty acid methyl esters (FAME)) of Grape seed oil (GSO).
